# Coupling Spatiotemporal Community Assembly Processes to Changes in Microbial Metabolism

**DOI:** 10.3389/fmicb.2016.01949

**Published:** 2016-12-16

**Authors:** Emily B. Graham, Alex R. Crump, Charles T. Resch, Sarah Fansler, Evan Arntzen, David W. Kennedy, Jim K. Fredrickson, James C. Stegen

**Affiliations:** Biological Sciences Division, Pacific Northwest National LaboratoryRichland, WA, USA

**Keywords:** niche, selection, dispersal, microbial community structure, aerobic respiration, ammonia oxidation, hyporheic, Hanford

## Abstract

Community assembly processes generate shifts in species abundances that influence ecosystem cycling of carbon and nutrients, yet our understanding of assembly remains largely separate from ecosystem-level functioning. Here, we investigate relationships between assembly and changes in microbial metabolism across space and time in hyporheic microbial communities. We pair sampling of two habitat types (i.e., attached and planktonic) through seasonal and sub-hourly hydrologic fluctuation with null modeling and temporally explicit multivariate statistics. We demonstrate that multiple selective pressures—imposed by sediment and porewater physicochemistry—integrate to generate changes in microbial community composition at distinct timescales among habitat types. These changes in composition are reflective of contrasting associations of *Betaproteobacteria* and *Thaumarchaeota* with ecological selection and with seasonal changes in microbial metabolism. We present a conceptual model based on our results in which metabolism increases when oscillating selective pressures oppose temporally stable selective pressures. Our conceptual model is pertinent to both macrobial and microbial systems experiencing multiple selective pressures and presents an avenue for assimilating community assembly processes into predictions of ecosystem-level functioning.

## Introduction

The collective effects of community assembly processes (e.g., dispersal, drift, and selection) on microbial metabolism of carbon and nutrients in the environment are poorly understood, and they constitute a key knowledge gap in process-based modeling of biogeochemical cycles. Selection and dispersal both have the potential to impact rates of microbial metabolism. For example, selection can enhance metabolism via species sorting mechanisms that optimize the microbiome for a given environment (Van der Gucht et al., 2007; Lindström and Langenheder, 2012), while dispersal limitation can inhibit immigration of metabolic diversity, and in some cases, lead to a maladapted and poorly functioning community (Telford et al., 2006; Lindström and Östman, 2011; Hanson et al., 2012; Peres et al., 2016). The extent to which community assembly processes regulate metabolism is contingent on myriad spatiotemporal dynamics including the geographic distance separating communities, the rate of environmental change, and historical abiotic conditions (Fukami et al., 2010; Graham et al., 2014; Nemergut et al., 2014; Hawkes and Keitt, 2015; Graham et al., 2016). Yet, we lack a conceptual basis for how multiple community assembly processes jointly influence microbial metabolism (Prosser et al., 2007; Gonzalez et al., 2012; Shade et al., 2013; Graham et al., 2016).

Community assembly processes act through space and time to impact community membership, which then impacts microbial metabolism (Vellend, 2010; Nemergut et al., 2013). For instance, communities experiencing a history of strong and consistent selection may contain taxa that are well-adapted to their environment and exhibit high metabolic rates. Alternatively, intense, unyielding selection may eliminate microbial taxa that metabolize scarce resource pools and impair community metabolic functioning (Grime, 1998; Loreau and Hector, 2001; Knelman and Nemergut, 2014). In this case, more diverse communities (e.g., those experiencing higher rates of dispersal or counteracting selective pressures) would be expected to exhibit higher and more consistent rates of metabolism than those structured by one dominant selective pressure. Dispersal limitation can inhibit the ability of organisms to reach their optimal environment, resulting in lower rates of community metabolism, while high rates of dispersal may either reduce or enhance microbial metabolism, respectively, by allowing for immigration of maladapted organisms or by increasing biodiversity (Hooper et al., 2012; Nemergut et al., 2014).

Additionally, individual taxa are differentially impacted by community assembly processes. Only some taxa contain traits that are under selection in given environmental conditions, and changes in the environment may affect some taxa to a greater extent than others (Poff, 1997; Lebrija-Trejos et al., 2010; Knelman and Nemergut, 2014; Krause et al., 2014). For example, a change in a certain nutrient should have a larger influence on taxa that directly metabolize it than those that utilize it as a secondary resource. Similarly, traits that facilitate dispersal (e.g., spore formation) are preferentially contained within certain taxa (Martiny et al., 2006; Tremlová and Münzbergová, 2007) such that changes in abiotic transport mechanisms should have contrasting effects on taxa with or without traits that facilitate dispersal. Thus, taxon-specific relationships between assembly processes and microbial communities may inform our understanding of the ecological processes influencing changes in environmental microbiomes beyond trends we observe at the community-level.

Environmental transition zones present a unique opportunity for examining interactions between microbial metabolism and both long- and short-term assembly processes, as they experience extreme spatiotemporal variation in physicochemical characteristics and microbial community composition across tractable spatial and temporal scales. Here, we leverage inherent variation in hydrology, habitat heterogeneity, and aerobic respiration in a zone of subsurface groundwater-surface water mixing (hereafter termed “hyporheic zone”) to examine the interplay of community assembly processes and microbial metabolism through time. Hyporheic zones are subsurface regions below and adjacent to rivers and streams that experience mixing between surface water and groundwater. The groundwater-surface water mixing within the hyporheic zone can result in blending of complementary resources and, in turn, elevated rates of microbial metabolism relative to other systems (Hancock et al., 2005; Boulton et al., 2010).

We employ null modeling in conjunction with temporally explicit multivariate statistics to characterize assembly processes driving shifts in microbial communities and microbial metabolism in the Columbia River hyporheic zone. We examine two co-occurring, yet ecologically distinct, habitat types—attached and planktonic communities. Further, we extend the analysis of community assembly processes to individual taxa that likely contribute to observed shifts in microbial metabolism. Our results culminate in a broadly applicable conceptual model coupling changes in selective environments, trait abundance, and ecosystem-level functioning through time.

## Materials and Methods

### Study Design

This study was conducted in Hanford Reach of the Columbia River adjacent to the Hanford 300A (approximately 46°22′ 15.80′′N, 119° 16′ 31.52′′W) in eastern Washington, as described elsewhere (Slater et al., 2010; Zachara et al., 2013; Stegen et al., 2016), from March to November 2014. The hyporheic zone of the Columbia River experiences geographic variation in groundwater-surface water mixing, porewater geochemistry, and microbial community composition on sub-hourly to annual timescales (Peterson and Connelly, 2004; Arntzen et al., 2006; Slater et al., 2010; Lin et al., 2012; Stegen et al., 2012, 2016; Zachara et al., 2013). Accordingly, the Hanford Reach of the Columbia River embodies a model system to facilitate the integration of community ecology and microbial metabolism.

We monitored physicochemical conditions for three hydrologically-connected geographic zones (nearshore, inland, river) via aqueous sampling (Table S1). The inland environment is characterized by an unconfined aquifer within the Hanford formation and more recent illuvial deposits, and it maintains a distinct hydrologic environment with stable temperatures (∼15°C) and high concentrations of anions and inorganic carbon relative to the river. River water contains high concentrations of organic material and low concentrations of ions with seasonally variable temperatures. The waters from these discrete hydrologic environments experience dynamic mixing in a nearshore hyporheic zone that is regulated by fluctuations in river stage; we focus on ecological dynamics within this zone. To monitor groundwater-surface water mixing across space and time, we utilize Cl^-^ as a conservative tracer for groundwater contributions to hyporheic porewater chemistry as employed by Stegen et al. (2016).

Detailed sampling and analytical methods are in the Supplementary Material. Attached and planktonic communities were obtained from deployed colonization substrate and aqueous samples in the hyporheic zone. Samples to construct the regional species pool for null models were simultaneously obtained at three inland wells and at one location in the Columbia River (*n* = 0-4 at each sampling event, a full breakdown of sample size is listed in Table S2). These samples were collected at three-week intervals from March through November 2014, with the first planktonic samples collected in March and the first attached samples collected after a 6-week incubation period from piezometers installed to 1.2 m depth near the riverbed. Samples collected at each time point were assumed to represent sub-hourly variation in hydrologic conditions, as hydrology fluctuates at sub-hourly rates within a single day in our system (Arntzen et al., 2006), whereas samples collected across the full sampling period were assumed to represent seasonal variation.

Aqueous samples were obtained by pumping water from piezometers adjacent to colonization substrates and used to derive physicochemical conditions as well as to sample planktonic communities. Attached microbial communities were sampled by deploying mesh stainless steel incubators of locally sourced colonization substrate in piezometers within one meter of piezometers from which aqueous samples were obtained. All incubators were deployed 6 weeks prior to removal. For each sample, we collected the following data according to procedures outlined in the Supplementary Material: physicochemical characteristics (aqueous samples only), microbial metabolism (aerobic metabolism assayed with Resazurin) per unit active biomass (ATP) defined by Raz:ATP (attached samples only), and 16S rRNA amplicon sequences (all samples). Sequences are publically available at doi: 10.6084/m9.figshare.4264148.

### Null Modeling Approach

We implemented null modeling methodology developed by Stegen et al. (2013, 2015) using *R* software^1^ to disentangle community assembly processes (Supplementary Material). The approach uses pairwise phylogenetic distances between communities, calculated using the mean-nearest-taxon-distance (βMNTD) metric (Webb et al., 2008; Fine and Kembel, 2011), to infer the strength of selection. Communities were evaluated for significantly less turnover than expected (βNTI < -2, homogeneous selection) or more turnover than expected (βNTI > 2, variable selection) by comparing observed βMNTD values to the mean of a null distribution of βMNTD values—and normalizing by its standard deviation—to yield βNTI (Stegen et al., 2012). Pairwise community comparisons that did not deviate from the null βMNTD distribution were evaluated for the influences of dispersal limitation and homogenizing dispersal by calculating the Raup-Crick metric extended to account for species relative abundances (RC_bray_), as per Stegen et al. (2013, 2015). Observed Bray-Curtis dissimilarities were compared to the null distribution to derive RC_bray_. RC_bray_ values > 0.95, > -0.95 and < 0.95, or < -0.95 were assumed to indicate dispersal limitation, no dominant assembly process, or homogenizing dispersal, respectively. Significance levels for βNTI and RC_bray_ are based on standard deviations—|βNTI| = 2 denotes two standard deviations from the mean of the null distribution—and alpha values—|RC_bray_| = 0.95 reflects significance at the 0.05 level. Inferences from both βNTI and RC_bray_ have previously been shown to be robust (Dini-Andreote et al., 2015; Stegen et al., 2015).

### Statistical Methods

Regressions and one-sided Mann Whitney *U* tests were conducted using the base statistics package in *R*. Variation in community composition was assessed with PERMANOVA in QIIME (Caporaso et al., 2010). We fit porewater characteristics to NMDS plots of Bray-Curtis dissimilarities with and without stratifying by time within attached and planktonic communities using the ‘vegan’ package in *R* (999 permutations, Oksanen et al., 2013). Further details are available in the Supplementary Material.

Because we observed large seasonal differences in species richness in both attached and planktonic communities, we performed similarity percentage analysis (SIMPER) in ‘vegan’ to identify individual species driving community dissimilarity (Clarke, 1993). SIMPER was conducted across time periods of high and low species richness within attached and planktonic communities separately (i.e., communities from sampling points with high vs. low richness in each habitat type). We grouped communities by sampling date (determined by the average species richness in communities at each time point) to control for seasonal effects. Further, we used SIMPER to identify species that differentiated attached and planktonic communities regardless of differences in richness (i.e., all attached communities vs. all planktonic communities).

We extracted taxonomic groups of organisms at the class-level containing at least one species identified as having a significant impact on community composition by SIMPER (*P* < 0.05) for subsequent analyses. Organisms were grouped at the class-level to provide sufficient statistical power for analysis. Mantel tests were used to compare the average relative abundance of taxonomic groups identified by SIMPER across samples to associated βNTI and RC_bray_ values (‘vegan’, 999 permutations). Finally, we compared dissimilarity in species richness between samples within and across attached and planktonic communities to βNTI and further, if -2 < βNTI < 2, to RC_bray_ using Mantel tests to infer community assembly processes generating species turnover between samples.

## Results

### Hydrologic and Community Shifts Through Time

**FIGURE 1:**
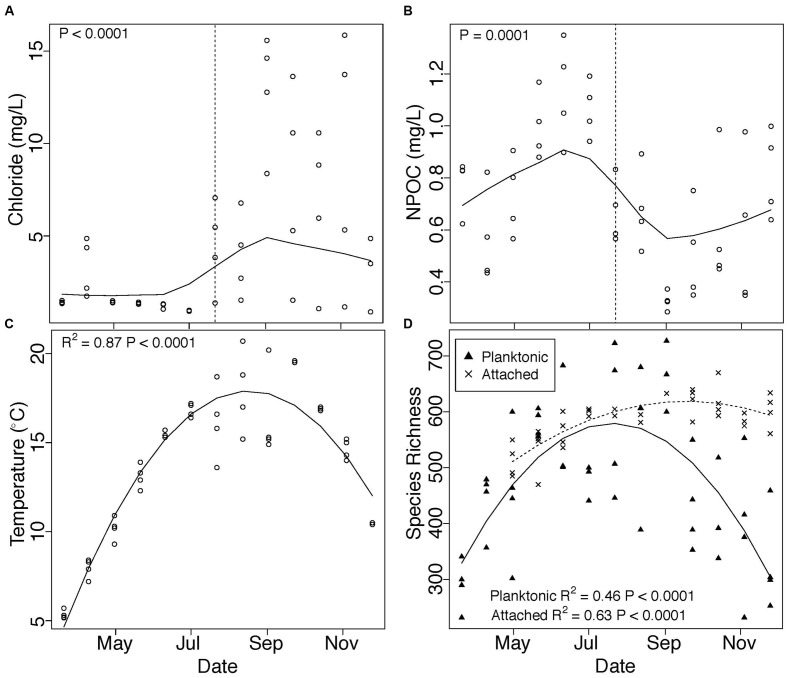
**Changes in (A)** chloride concentration, **(B)** NPOC concentration, **(C)** temperature, and **(D)** species richness across our sampling period are depicted in **Figure 1**. Chloride and NPOC concentration show abrupt shifts beginning at our July 22 sampling point (vertical dashed lines). *P*-values in **(A)** and **(B)** denote one-sided Mann-Whitney *U* test results of samples taken before versus on or after July 22, while trends through time in **(A)** and **(B)** are displayed using locally weighted scatterplot smoothing (LOWESS). Quadratic polynomials were fit to temperature and species richness data and plotted in **(C,D)**. Triangles in **(D)** represent planktonic communities; X’s represent attached communities.

We observed distinct temporal trends in microbial communities and in groundwater-surface water mixing, characterized by an abrupt increase in Cl^-^ concentration (**Figure 1A**) and decrease in NPOC (**Figure 1B**) associated with a seasonal shift in water stage (Figure S1 and Table S1). Temperature peaked during August and followed a smooth temporal trend (**Figure 1C**). The composition of both attached (PERMANOVA, *R*^2^ = 0.44, *P* = 0.001) and planktonic (PERMANOVA, *R*^2^ = 0.44, *P* = 0.001) communities also changed across our sampling period, but attached and planktonic communities remained taxonomically distinct through time (PERMANOVA, *R*^2^ = 0.19, *P* = 0.001). Main taxa in each environment are presented in Table S3.

**FIGURE 2:**
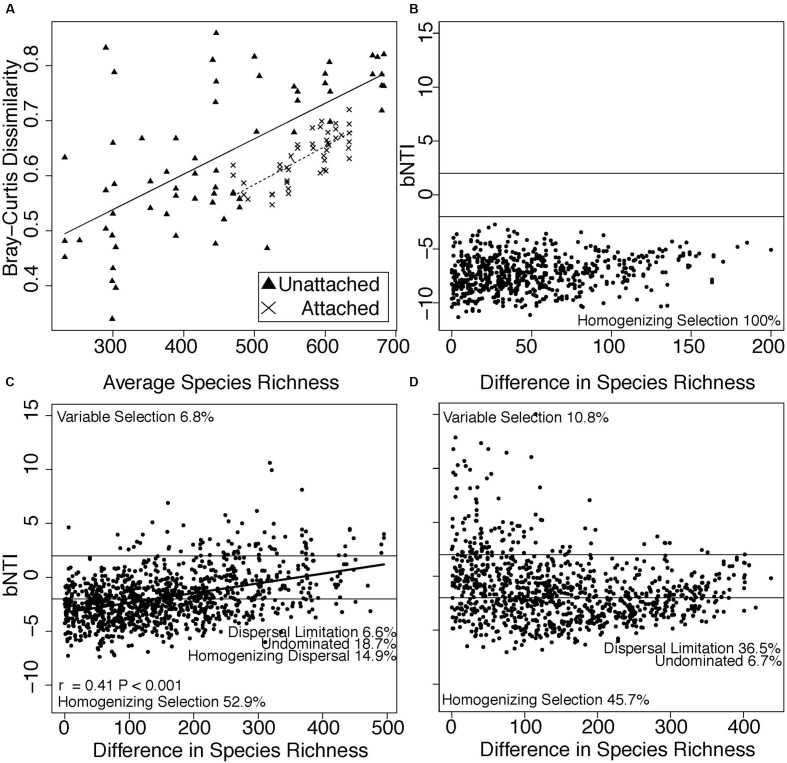
**(A)** Bray-Curtis dissimilarity within each sampling time point increased as mean species richness increased in attached (X’s) and planktonic (triangles) communities. In addition, βNTI values across differences in species richness are shown for **(B)** attached, **(C)** planktonic, and **(D)** attached vs. planktonic communities. Horizontal lines at βNTI = -2 and βNTI = 2 denote thresholds for assembly processes. βNTI values less than -2 suggest assembly is governed by homogeneous selection, while values greater than 2 suggest assembly is governed by variable selection. Stochastic assembly processes (dispersal limitation, homogenizing dispersal) and undominated assembly processes lie between βNTI -2 and 2. The proportion of βNTI values within each category are listed as text in **(B-D)**. A linear regression trend line is depicted in **(C)** with significance assessed via Mantel test.

Species richness in both attached and planktonic communities mirrored temperature trends, with the highest number of species observed during the warmest summer months (**Figure 1D**). Richness was more tightly correlated with temperature (Figure S2, regression, attached: *R*^2^ = 0.25, *P* = 0.001, planktonic: *R*^2^ = 0.22, *P* = 0.002) than Cl^-^ (regression, attached: *P* = 0.01, *R*^2^ = 0.14, planktonic: *P* > 0.05) and NPOC (regression, attached: *P* = 0.04, *R*^2^ = 0.10, planktonic: *P* > 0.05). Finally, within both environments, community dissimiliarity increased in concert with differences in species richness (**Figure 2A**).

### Spatiotemporal Assembly Processes

Dissimilarity in microbial communities indicated a possible role for different assembly processes governing species composition among (i.e., within attached and planktonic separately) and across (i.e., attached vs. planktonic) habitats. We investigated assembly processes across differences in richness to identify processes that impacted species addition to each habitat, and we found that assembly varied between habitats (**Figures 2B,C**). βNTI was positively correlated with differences in species richness in planktonic samples (Mantel, *P* = 0.001, *r* = 0.41, **Figure 2C**), with weaker correlations in attached communities (Mantel, *P* = 0.02, *r* = 0.24, **Figure 2B**) and between attached and planktonic communities (Mantel, *P* = 0.006, *r* = -0.26, **Figure 2D**).

**FIGURE 3:**
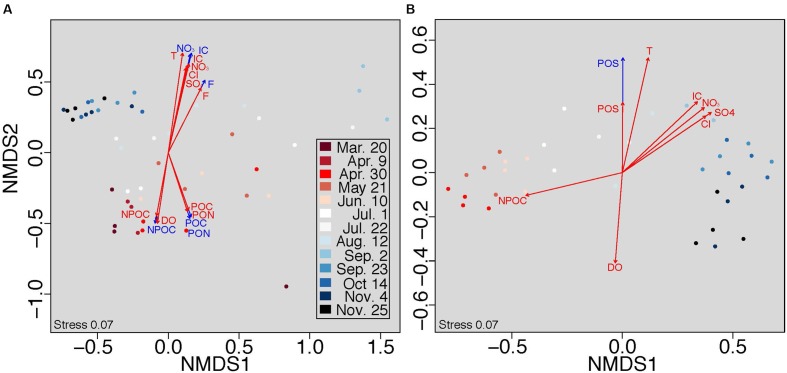
**Non-metric multidimensional scaling (NMDS) analysis was conducted on Bray-Curtis distances within (A)** planktonic and **(B)** attached communities. Colors denote seasonal shifts in community structure along a gradient from March (red) to November (blue). Physicochemical characteristics were fit to each plot with (blue arrows) and without (red arrows) stratifying permutations by sampling time to assess short- and long-term community responses, respectively, to the aqueous environment.

Because selection was a substantial driver of all microbial communities, we further examined the impact of aqueous physicochemistry on communities at both sub-hourly (within sampling date) and seasonal (across all sampling dates) timescales using stratified and unstratified NMDS analysis. Permutations in stratified NMDS are constrained within the specified grouping (in our case, sampling date), thus controlling for the grouping factor (see Oksanen et al., 2013). Here, we infer significant relationships when stratifying by time to represent dynamics occurring within each sampling date and those occurring in analyses without stratification to represent dynamics across sampling dates. Planktonic communities were correlated to more environmental variables than attached communities at a sub-hourly timescale (NMDS stratified by time, **Figures 3A,B**; Table S4). Conversely, attached communities correlated more tightly with physicochemical attributes over a seasonal timescale (unstratified NMDS, **Figures 3A,B**).

### Phylogenetic Variability in Assembly and Associated Shifts in Metabolism

**FIGURE 4:**
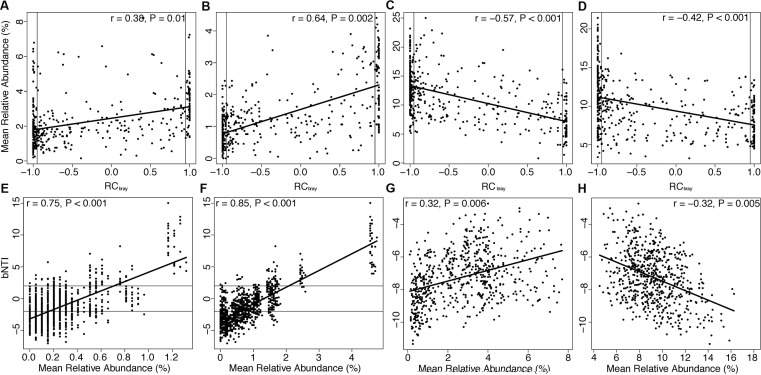
**Relationships between βNTI or RC_bray_ and the mean abundance (across samples) of selected taxa identified by SIMPER analysis are depicted in Figure 4. (A-D)** Demonstrate relationships of *Thaumarchaeota*, *Acidobacteria-6*, *Actinobacteria*, and *Alphaproteobacteria*, respectively, versus RC_bray_ in planktonic communities. Horizontal lines at βNTI = -2 (homogeneous selection) and βNTI = 2 (variable selection) and vertical lines at RC_bray_ = -0.95 (homogenizing dispersal) and 0.95 (dispersal limitation) denote thresholds for assembly processes. Trend lines in all panels were derived from linear regressions and significance was assessed via Mantel test. **(E,F)** Show relationships of βNTI with *Parvarchaeota* and *koll11* in attached vs. planktonic communities; while **(G,H)** denote relationships of βNTI with *Thaumarchaeota* and *Betaproteobacteria* within attached communities, respectively.

Similarity percentage analysis revealed species driving differences among attached and planktonic communities during periods of high versus low species richness (Table S5). We extracted phylogenetic classes of organisms containing species identified by SIMPER and examined relationships between their relative abundances and βNTI and RC_bray_. All significant correlations with absolute *r* values greater than 0.30 are listed in Table S5. Taxa in planktonic communities exhibited no relationships with βNTI (Table S6), but the mean relative abundance of many taxa, including *Thaumarchaeota* (positive, **Figure 4A**), a class of *Acidobacteria* (positive, **Figure 4B**), *Actinobacteria* (negative, **Figure 4C**), and *Alphaproteobacteria* (negative, **Figure 4D**), displayed correlations with RC_bray_. The mean relative abundance of *Parvarcheota* and a class of candidate phyla OP3 (*koll11*) were positively correlated with βNTI derived from comparisons across attached and planktonic communities (**Figures 4E,F**). Finally, within attached communities, βNTI was correlated with the mean relative abundance of *Thaumarchaeota* (positive, **Figure 4G**) and *Betaproteobacteria* (negative, **Figure 4H**). No correlations were observed between RC_bray_ and taxa within attached communities or across attached-vs.-planktonic communities (Table S6).

**FIGURE 5:**
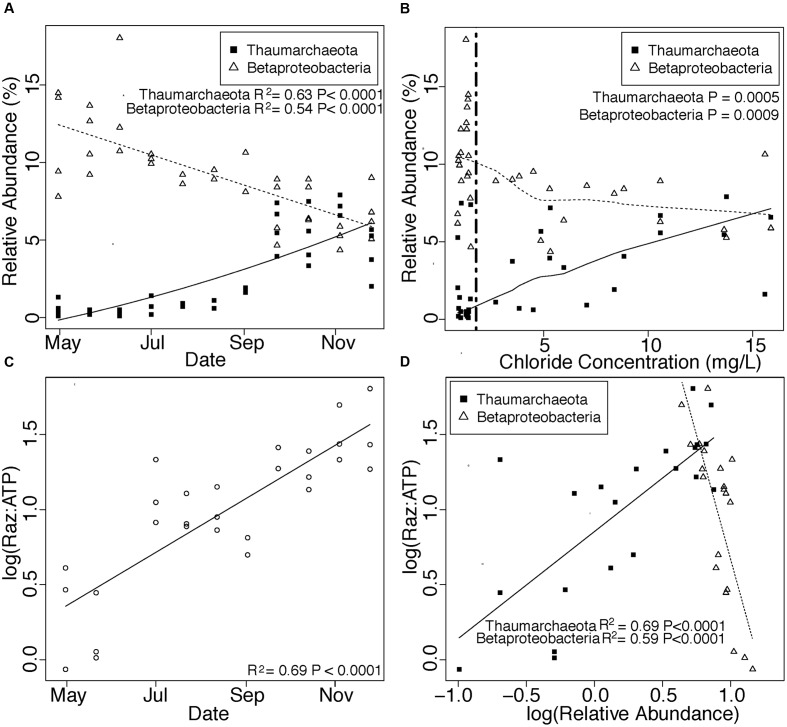
**(A,B)** Changes in *Thaumarchaeota* and *Betaproteobacteria* across changes in time **(A)** and chloride concentration **(B)**. Trend lines in **(A)** denote linear (*Betaproteobacteria*) and quadratic (*Thaumarchaeota*) regressions. The vertical line and statistics in **(B)** denote one-sided Mann-Whitney *U* test results of *Betaproteobacteria* and *Thaumarchaeota* when chloride concentrations are above or below the maximum Cl^-^ concentration in the Columbia River (1.83 mg/L). **(C)** Shows increases in aerobic respiration normalized to active biomass (Raz:ATP) through time. Finally, **(D)** shows relationships of *Betaproteobacteria* and *Thaumarchaeota* with Raz:ATP. Trend lines and associated statistics in **(C,D)** were derived with linear regressions. *Thaumarchaeota* and *Betaproteobacteria* are shown as closed squares and open triangles, respectively, in **(A,B,D)** with trends for each group shown with a solid (*Thaumarchaeota*) or dashed (*Betaproteobacteria*) line.

We also observed a seasonal increase in the abundance of *Thaumarchaeota* in attached communities and a concomitant decrease in *Betaproteobacteria* (**Figure 5A**) that corresponded with shifts in hydrology (**Figure 5B**). Oxygenated conditions persisted throughout our sampling period, and Raz:ATP increased seasonally within attached communities, an effect that correlated with day of year (**Figure 5C**) but not temperature, NPOC concentration, or hydrology (regression: temperature *P* = 0.10, NPOC *P* = 0.21, log(Cl^-^) *P* = 0.15). The relative abundance of *Thaumarchaeota* and *Betaproteobacteria* in attached communities also correlated positively and negatively, respectively, with Raz:ATP (**Figure 5D**) and exhibited contrasting responses to porewater physicochemical properties (Table S7).

## Discussion

Our results show pronounced seasonal changes in hydrologic and microbial characteristics within the Columbia River hyporheic zone, as well as variation in the importance of selection and dispersal in structuring attached vs. planktonic communities. These community assembly processes operated at distinct timescales in each habitat and were also associated with changes in the relative abundance of certain taxa. In particular, changes in selection exhibited contrasting relationships with putative heterotrophic and autotrophic taxa in attached communities. Further, changes in community-level microbial metabolism correlated with the abundance of these same taxa in attached communities. Based on our findings, we present a conceptual model applicable within both macrobial and microbial systems that links trait selection, organismal fitness, and ecosystem-level functioning in habitats that are characterized by opposing selective pressures across multiple timescales.

### Microbial Responses to Environmental Change

Hyporheic microbial community composition shifted in conjunction with seasonal changes in organic carbon concentration, temperature, and groundwater-surface water mixing conditions. These variables each explained some variation in temporal community dissimilarity within attached and planktonic communities, indicating potential influences of selection (e.g., mediated by environmental change) and dispersal (e.g., mediated by hydrologic transport) over microbial community composition (Table S8). Both selection by the geochemical environment and dispersal from local sediment communities have been demonstrated within the groundwater aquifer in our system (Stegen et al., 2012); however, the balance of these two processes in structuring hyporheic microbial communities remains unclear.

Our results indicate that homogeneous selection (i.e., consistently imposed selection for a given set of traits, βNTI < -2) was the dominant assembly process in attached communities, while planktonic communities were influenced by a combination of homogeneous selection, variable selection (i.e., selective pressures that change through space or time, βNTI > 2), and spatial processes (i.e., dispersal, -2 < βNTI < 2 and |RC_bray_| > 0.95). Because planktonic communities consistently displayed higher βNTI values in comparison to attached communities, often with -2 < βNTI < 2, dispersal processes may play a greater role in structuring planktonic vs. attached communities. In contrast, attached communities always yielded large negative βNTI values suggesting that features of the physical sediment environment may impose stronger selective pressures than aqueous physicochemistry.

Because we observed pronounced seasonal trends in species richness associated with changes in the environment, we used null modeling to assess the extent to which selection versus dispersal influenced community composition across changes in species richness. Homogenous selection prevailed in attached communities as differences in species richness increased (**Figure 2B**), indicating strong and consistent selective pressures imposed by a relatively stable environment across temporal changes in microbial diversity. Strong consistent selection through time in attached communities suggests that the physical substrate may inherently contain a limited number of ecological niches—potentially related to mineralogy or physical structure—with slow changes in available niche space. In this case, temporal increases in richness were likely due to the addition of taxa that were ecologically similar to existing taxa and occupied similar niche space.

In contrast, differences in planktonic species richness were positively correlated with βNTI, with βNTI values supporting a role for more variable selection as differences in richness increased (**Figure 2C**). In this case, increases in species richness were likely due to the addition of taxa occupying newly available niche space generated by changes in the selective environment, yielding new taxa that were ecologically dissimilar to existing taxa.

Variation in assembly processes between attached and planktonic communities may be due to inherent differences among these environments, such as influences of mineralogy (Carson et al., 2007; Jorgensen et al., 2012), physical matrix composition (Vos et al., 2013; Breulmann et al., 2014), and/or relative rates of change in environment characteristics (discussed below). Further, differences in assembly processes and niche dynamics between the attached and planktonic communities may also be reflective of differing rates of organismal response to fluctuations in the hyporheic environment.

### Timescales of Selection

The timescales at which selection imposes constraints on microbial community composition are poorly understood (Shade et al., 2012; Nemergut et al., 2013). Here, we provide new insights into these timescales within the hyporheic zone, showing that selection on planktonic communities operates at the timescale of shifting porewater conditions (sub-hourly to seasonal), while selection on attached communities operates primarily at seasonal timescales (**Figure 3**). Both communities experienced a seasonal change in composition; however, variation in planktonic communities correlated with porewater physicochemistry at both the sub-hourly and seasonal timescales (**Figure 3A**, red and blue arrows, respectively). In our system, planktonic organisms may therefore be influenced by short-term fluctuations in groundwater-surface mixing, either through rapid changes in the selective environment or dispersal via hydrologic transport.

In contrast, selection on attached communities was detectable only at the seasonal timescale and exhibited resistance to short-term hydrologic fluctuations (**Figure 3B**). This short-term stability could be facilitated by a number of potentially complementary mechanisms. For example, attached communities may reside within biofilms, whereby microbial cells are imbedded in a matrix of extracellular polymeric substances. Biofilms are prevalent in aquatic systems and buffer communities against fluctuations in the hydrologic environment (Battin et al., 2016). Attached microbial communities may also have adhesion mechanisms (Hori and Matsumoto, 2010) that confer stability. Community assembly processes, such as priority effects, may also contribute to relatively slow rates of community turnover (Fukami, 2004; Fukami et al., 2010). Attached communities contained more species (on average) than planktonic communities (**Figure 1D**), further suggesting that temporal stability in attached communities may be enhanced by high species richness reducing susceptibility to invasion (Stachowicz et al., 2002).

### Taxon-Specific Assembly Processes

Taxon-specific assembly processes are masked when relating βNTI and RC_bray_ to environmental variables. To elucidate taxon-specific selection and dispersal mechanisms, we compared the relative abundance of taxa identified by SIMPER to βNTI and RC_bray_ values.

In light of rapid hydrologic fluctuations in the porewater environment, relationships between RC_bray_ and taxa abundances in planktonic communities provide evidence for a role of taxon-specific dispersal mechanisms (Table S6). In particular, positive relationships of *Thaumarchaeota* (**Figure 4A**) and a class of *Acidobacteria* (**Figure 4B**) with RC_bray_ and negative relationships of *Actinobacteria* (**Figure 4C**) and *Alphaproteobacteria* (**Figure 4D**) with RC_bray_ were among the strongest correlations (Table S6). These positive or negative relationships indicate higher or lower relative abundances, respectively, under higher levels of dispersal limitation. No relationships existed between planktonic taxa abundances and βNTI.

Although we cannot be certain of the mechanisms responsible for RC_bray_-abundance relationships in planktonic communities, the trends we observed help elucidate ecological dynamics impacting the abundance of microbial taxa within hyporheic zones. For example, taxa showing positive relationships with RC_bray_—*Acidobacteria* and *Thaumarchaeota*—are widely distributed globally (Francis et al., 2005; Fierer and Jackson, 2006; Jones et al., 2009; Pester et al., 2011), suggesting these organisms may be able to disperse under community-level dispersal limitation. Conversely, taxa exhibiting negative relationships with RC_bray_ have physiologies that may diminish dispersal ability. *Alphaproteobacteria* can produce filaments that aid in attachment (Kragelund et al., 2006; Jones et al., 2007), and dispersal limitation has been demonstrated in soil *Actinobacteria* (Eisenlord et al., 2012). Thus, negative relationships between these taxa and RC_bray_ may reflect an enhanced ability of these organisms to persist locally relative to other community members.

Additionally, when we examined assembly processes governing differences between attached and planktonic communities, we observed selection for microbial taxa in planktonic communities with unique ecological properties (Table S6). No correlations with RC_bray_ were found in these comparisons; however, we identified positive relationships between βNTI and the average relative abundance of two classes of organisms—a candidate class of archaea (*Parvarchaeota*, **Figure 4D**) and a class of the candidate phylum *OP3* (*koll11*, **Figure 4E**). Positive correlations between a particular taxon and βNTI across habitat types imply that the taxon increases in abundance as the habitats diverge in selective environments (i.e., selection becomes more variable).

In our system, *Parvarchaeota* and *koll11* were almost exclusively found in planktonic communities suggesting that selection in the porewater environment favors these organisms. Although the specific selective pressures regulating the abundance of these organisms are unknown, archaea and members of the PVC superphyla to which *OP3* belongs have a cell membrane lacking peptidoglycan that conveys resistance to common antibiotics and have the genetic potential to metabolize C1 compounds such as methane (Fuerst and Sagulenko, 2011). The distinctive features of these organisms and abundance within our system merits future investigating into their role in carbon cycling in hyporheic environments.

Finally, we observed changes in the abundance of two major taxa—*Betaproteobacteria and Thaumarchaeota*—within attached communities that correlated with changes in βNTI. Members of *Betaproteobacteria* increased in relative abundance with increases in the strength of homogeneous selection (**Figure 4H**), which occurred during times of low groundwater intrusion (**Figures 5A,B**). In contrast, members of *Thaumarchaeota* (**Figure 4G**) increased as homogeneous selection waned during times of high groundwater intrusion (**Figures 5A,B**). In this scenario positive correlations between a taxon and βNTI indicate that the taxon becomes more abundant as homogeneous selection wanes, and thus, that selection targets traits outside the taxon. In contrast, negative correlations between a taxon and βNTI should indicate that the primary selective pressure is for traits contained within that taxon. Because organisms with wider niche breadths are favored by selection in a greater variety of environmental conditions, positive relationships should be more probable for organisms occupying niches defined by a narrow subset of environmental attributes, whereas negative relationships should be more probable for organisms occupying broader niches. Indeed, *Betaproteobacteria* and *Thaumarchaeota*, respectively, contain organisms with diverse and limited metabolic capabilities (Amakata et al., 2005; Yang et al., 2005; Sato et al., 2009; Pester et al., 2011; Beam et al., 2014; Weber et al., 2015).

### Functional Effects Through Time

Shifts in the relative abundances of *Betaproteobacteria* and *Thaumarchaeota* also correlate with changes in groundwater-surface water mixing and in rates of microbial metabolism, denoting a link between community assembly processes and seasonal trends in autotrophic vs. heterotrophic metabolism. *Betaproteobacteria* is a metabolically diverse taxon, exhibiting a range of aerobic and facultative metabolisms including methylotrophy (Kalyuzhnaya et al., 2006), ammonia-oxidation (Freitag et al., 2006), nitrogen fixation (Rees et al., 2009), phototrophy (Gifford et al., 2013), and a variety of heterotrophic metabolisms (Amakata et al., 2005; Yang et al., 2005; Sato et al., 2009). Although we cannot be certain of the primary metabolic role(s) of these organisms, a positive correlation between their abundance and NPOC concentration supports their important contribution to heterotrophy in this system (Table S7). *Betaproteobacteria* remained in high abundance relative to other organisms throughout our study period, despite a seasonal decline, possibly indicating a broad metabolic role for these organisms. In contrast, metabolic activity of *Thaumarchaeota* is primarily constrained to ammonia-oxidation (Pester et al., 2011; Beam et al., 2014; Weber et al., 2015). *Thaumarchaeota* abundance was negatively correlated with NPOC, consistent with their involvement in ammonia-oxidation (Table S7). The relative abundance of *Thaumarchaeota* also correlated with physicochemical properties associated with increased groundwater-surface water mixing (SO_4_^2^, NO^3^, IC, Table S7), suggesting a heightened importance of *Thaumarchaeota*-mediated nitrification when enhanced groundwater discharge into the hyporheic zone leads to organic carbon limitation (Taylor and Townsend, 2010).

Taken together, we hypothesize that selective pressures, both from sediment composition and from porewater physicochemistry, favor diverse heterotrophs within *Betaproteobacteria*. However, when the porewater environment changes due to a seasonal change in groundwater-surface water mixing, selective pressures shift to facilitate ammonia-oxidizing *Thaumarchaeota*. More broadly, we propose that community composition in dynamic environments is often the product of multiple selective pressures that operate across different timescales, resulting in an increase of specialist organisms during periods in which selection by an oscillating environment (e.g., hydrologic change) opposes that of a temporally stable environment (e.g., consistent sediment chemistry).

Importantly, changes in community composition in our system are associated with a seasonal increase in microbial metabolism, consistent with work in both micro- and macroecology demonstrating that productivity increases with niche diversification (Hooper et al., 2005; Cardinale et al., 2007; Cardinale, 2011; Gravel et al., 2011; Hunting et al., 2015). Specifically, we observed positive correlations between Raz:ATP and *Thaumarchaeota* abundance coincident with negative correlations between Raz:ATP and *Betaproteobacteria* (**Figure 5D**). We therefore infer that seasonally fluctuating selective pressures from the porewater environment impact microbial metabolism via their influences on niche dynamics. We propose that community-level niche diversification generated by a seasonal rise of specialized autotrophs leads to an increase in community metabolic activity despite selection for heterotrophs imposed by the consistent sediment environment.

### Ecological Implications

**FIGURE 6:**
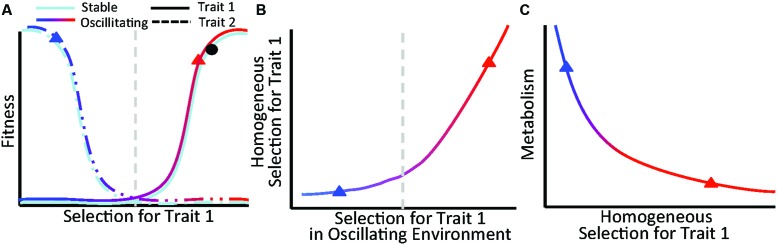
**Figure depicts a conceptual model describing relationships between trait selection, organismal fitness, and microbial metabolism for communities experiencing dual selective pressures. (A)** Selection for a trait follows a continuous gradient within a stable environment (light blue) and oscillating environment (blue to red gradient). Organisms that contain opposing traits (dashed vs. solid lines) are favored at each end of the spectrum, delineated here as to the left (selection against trait 1 and for trait 2) or right (selection for trait 1 and against trait 2) of the vertical gray line. Given selection in a stable environment denoted by the black dot in **(A)**, variation in homogeneous selection **(B)** is driven by the magnitude and direction of selection in the oscillating environment. When selection in the oscillating environment opposes selection in the stable environment, homogeneous selection decreases **(B)** and microbial metabolism increases **(C)** due to an increase in realized niche space and biodiversity. Blue and red triangles in **(B,C)** correspond to oscillating selection locations on the fitness landscape in **(A)**.

Our findings lead to a conceptual model describing relationships between trait selection, organismal fitness, and ecosystem functioning for communities experiencing multiple selective pressures (**Figure 6**). The conceptual model focuses on the combined influences of stable and oscillating selective pressures, which should be prevalent across ecosystems. For example, in terrestrial ecosystems, physical soil properties and soil water content are relatively stable and oscillating, respectively, from the perspective of associated plant communities. Likewise, in subsurface systems (such as presented here), sediment geochemistry is relatively stable over monthly timescales and hydrologic conditions are continuously fluctuating generating dual selective pressures for benthic organisms.

We specifically hypothesize that selection imposed by a stable feature of the environment can favor organisms possessing traits that oppose traits selected for by the oscillating environment (**Figure 6A**). Because the stable environment applies consistent selective pressures, shifts in the strength of homogeneous selection are driven by changes in the oscillating environment. For instance, given a stable environment represented by the black dot in **Figure 6A**, the strength of homogeneous selection in **Figure 6B** is influenced by selection imparted by the oscillating environment, denoted as a gradient from blue to red. In this scenario, increased selection from the oscillating environment causes a decrease in homogeneous selection that results in niche diversification and enhanced microbial metabolism (**Figures 6B,C**).

## Conclusion

While future work is needed to validate our conceptual model, our research represents a key step forward in spatiotemporal ecological research by assimilating shifts in community composition, assembly processes, and microbial metabolism. We develop this model by repeatedly sampling attached and planktonic microbial communities within an environmental transition zone over a 9-month time period. Our results indicate a rise in specialist autotrophs associated with increased rates of microbial metabolism when the direction of selection imposed by an oscillating hydrologic environment opposes that of stable selective pressures imposed by sediments. Further, we show that assembly processes operate at different temporal scales in planktonic and attached communities and have taxon-specific effects within each community. Our conceptual model can be applied in a range of ecological contexts beyond microbiology-for example, as a framework for understanding relationships between environmental change and competition under the Stress-Dominance hypothesis in macroecology (Brown et al., 1996; Normand et al., 2009) and for developing predictive models of species distributions under novel climate scenarios. As a whole, our work is an advancement in the integration of individual and community-level ecology theory with ecosystem function and develops a conceptual framework for coordinating assembly processes, changes in species abundance, and predictions of ecosystem-level functioning in response to environmental change.

## Author Contributions

EG was responsible for analyzing all data and writing with guidance from JS and JF. All other authors contributed substantially to experimental design, data collection, and manuscript revisions.

## Conflict of Interest Statement

The authors declare that the research was conducted in the absence of any commercial or financial relationships that could be construed as a potential conflict of interest.

## Footnotes

1 http://cran.r-project.org/
