# Supplementary material for: Coupling Spatiotemporal Community Assembly Processes to Changes in Microbial Metabolism
Source: Front Microbiol. 2016 Dec 16;7:1949. doi: 10.3389/fmicb.2016.01949 (PMC5226446; doi:10.3389/fmicb.2016.01949)
Supplement: Supplementary file 1 [file Data_Sheet_1.docx]

**Supplemental Material**

*Materials and Methods*

Samples were collected at three-week intervals near Hanford 300A from March through November 2014. This sampling schedule encompassed the elevated river stages in the late spring, when surface water intrusion into groundwater occurs, and the subsequent decline in river stage during late summer and fall. The nearshore hyporheic zone was sampled from 4 fully screened stainless steel piezometers as described below and form the basis of all analyses (1.2m screen length and 5.25cm inside diameter (MAAS Midwest, Huntley, IL)).

In addition, microbial communities for null model realizations were derived from inland groundwater and river samples obtained simultaneously and processed in conjunction with nearshore hyporheic zone samples. The inclusion of these samples accounts for dispersal of microorganisms into the nearshore zone via nearby hydrologically interconnected geographic areas. Inland samples were obtained from 4 inland monitoring wells, located approximately 250 meters from the riparian zone. These wells are screened across the top of the subsurface aquifer. River water was sampled adjacent to the piezometers. Aqueous samples from the nearshore and river zones were taken using a peristaltic pump, and the inland wells sampled using a submersible pump.

*Sampling of attached communities*

Sand microcosms were constructed using ~80 cm^3^ Hanford formation medium grade sand (>0.425mm <1.7mm, Central Pre-Mix, Pasco, WA) in a modified 2 x 4.5”, 18/8 mesh stainless steel infuser (R.S.V.P. International Inc. Seattle, WA) plugged with Pyrex fiber glass (Corning Inc. Corning, NT) and combusted at 450^o^C for 8hr. Sand packs were deployed in pairs within the wells and piezometers every 3 weeks, with sets being harvested after 6 weeks, such that two pairs had overlapping incubation periods.

Upon collection, sand packs were pooled into a combusted stainless steel pan (450^o^C for 8hr) and homogenized with a combusted stainless steel scoop before subsampling approximately10mL into each of 4 50-mL polypropylene tubes (Corning Inc. Corning, NY) for DNA analysis. These tubes were placed in whirl-pak bags (Nasco WHIRL-PAK, Salida, CA) and flash frozen in a dry ice/ethanol bath before being stored in the field on dry ice, and in the lab at -80^o^C. Four ~ 3mL sand subsamples were collected for resazurin reduction incubations with a sterile cutoff 5-mL syringe (Becton Dickinson Co. Franklin, NJ) into pre-weighed, 20-mL amber vials (Thermoscientific Miami, OK). Then, 3mL filtered water was added, and the vials were stored on ice and later in the lab at 4^o^C. Triplicate ~0.2cc sand subsamples were collected for ATP measurements with a sterile cutoff 1-mL syringe and preserved in 0.5mL 20mM EDTA (pH 7.4) in 2-mL cryovials, frozen on dry ice and stored at -20^o^C. Remaining sand in the tray was transferred to a 50-mL polypropylene tube with a sterile plastic scoop and preserved as previously described for DNA samples

*Aqueous Sampling*

At each well location, pumps were deployed and lines purged with water for 15 min. Flow rates, purge volumes pump controller settings were recorded. After initial purge and prior to the installation of Sterivex filters, the manifold was purged water for ~1 min. At each piezometer location, peristaltic pumps were deployed and manifolds purged with water for 10-15 minutes. At both well and piezometer locations water was pumped through 0.22 µm polyethersulfone Sterivex filters (Millipore Co. Billerica, MA) for 30 minutes. Sterivex filters were removed from the manifold and retained water was pushed through with a sterile 30-mL syringe before storage on dry ice in Whirl-pak bags and later at -80^o^C. Sterivex filtered water was used sampled for water chemistry.

Three samples for anions, dissolved organic, and inorganic carbon (NPOC, DIC) were collected into pre-cleaned 40-mL glass vials (Thermoscientific, Rockwood, TN) at 10-minute intervals, and stored at 4^o^C. These samples were split for ion chromatographic and carbon analysis in the lab. One cation sample was collected in a pre-acidified 15-mL polypropylene tube (Becton Dickinson Co., Franklin, NJ) and stored at 4^o^C. Handheld meters were used to measure water temperature and conductivity (Ultrameter II, Myron L Co Carlsbad, CA). Dissolved oxygen and temperature were measured with a YSI Pro ODO handheld with an optical DO probe (YSI Inc. Yellow Springs, OH). Unfiltered water was passed through pre-combusted 47-mm glass-fiber filters (Whatman, GE Healthcare, UK) for quantification of particulate organic carbon (POC). Water volume filtered for POC was quantified, with a target volume of 5L. Filters were stored at -20^o^C in plastic petri-dishes.

*Analytical methods*.

POC samples were removed from the freezer and air dried in a biological safety cabinet for ~72hr. To remove inorganic carbon, filters were placed on pre-baked watch glasses in a glass dessicator with a beaker of 5-10mL concentrated HCl, and sealed under light vacuum for 24hrs (Hedges and Stern, 1984). Filters were air-dried in a fume hood overnight then wrapped in 40 x 40 mm silver foil (Elementar Co, Germany). The silver foil covered POC filters were then wrapped in 50 x 50mm squares of pure tin foil (Elemental Microanalysis, UK) and compressed to remove air with a manual pressing tool (Elementar Co.). POC samples were analyzed for C, N & S with an Elementar vario EL cube.

ATP assays were performed on samples derived from sandpacks using a Bac Titer-Glo cell viability assay kit (Promega, Madison, WI.). Samples were thawed at 4^o^C, weighed, and then equilibrated to room temperature for 1hr prior to analysis. 10mL ATP assay reagent components were thawed at 4^o^C overnight, brought up to room temperature for 1hr, mixed and equilibrated for 2hr prior to use. ATP standards were made in 20mM EDTA from a 10mM stock (Promega) in sterile 1.7-mL microfuge tubes (Genemate Bioexpress) and stored at -20^o^C. Raw luminescence in samples was measured using a Modulus (Turner Biosystems, Sunnyvale, CA) fitted with a luminescence module. Sand subsamples were assayed by adding 0.5mL ATP reagent and 25μl 0.4M MgCl_2_ to the cryovials, vortexing for 10 seconds, and then incubating for 5 minutes before measuring luminescence on a 200μl subsample.

Samples collected for resazurin reduction assays (Raz, sandpack only) were stored overnight at 4^o^C. One replicate vial from each location was heat killed in a 72^o^C, 140rpm shaking water bath for 30 minutes, and cooled on ice to bring back to 4^o^C. Resazurin incubations were started by adding 100μl of 30μM resazurin to vials when cold, gently mixing and then incubating on an angle at 50rpm and 21^o^C. After 48hr, vials were weighed and 3mL acetonitrile (ACN) added to begin a 1hr extraction. After ACN addition, vials were sealed, vortexed and weighed again before being placed in a sonicator bath for 10min. After sonication, vials were put back on the 50rpm shaker. After the 1hr extraction, vials were vortexed and sand was allowed to settle. The screw cap was removed to allow extract to be drawn into a 5-mL syringe fitted with a 20G needle. Extracts were filtered with 33mm, 0.2μm syringe filters (PES, Millex by Millipore) into pre-labeled 12-mL amber vials (Thermoscientific) and stored at 4^o^C. The vial with the remaining sand was dried in a convection oven at 75 ^o^C for at least 72 hrs then weighed. Fluorescence emission maxima for resazurin (630nm) and resorufin (585nm) were measured on resazurin sample extracts using a Horiba Fluorolog 3 fluorimeter. 2mL extract was added to 0.2mL 100mM HEPES (pH 8) in quartz cuvettes and fluorescence intensity quantified by comparison to resazurin and resorufin standards made up in ACN:H_2_O (1:1).

Dissolved inorganic carbon (DIC) and non-purgeable organic carbon (NPOC) in aqueous samples were determined by combustion catalytic oxidation/NDIR method using a Shimadzu TOC-Vcsh with ASI-V auto sampler (Shimadzu Scientific Instruments, Columbia, MD). The DIC was determined by injection into 25 % phosphoric acid at ambient temperature. NPOC was determined by acidification of the sample with 2 N HCl with a 5 minute sparging time to remove DIC. The sample was then injected into the furnace set to 680^o^C. A Dionex ICS-2000 anion chromatograph with AS40 auto sampler was used to determine nitrate and chloride concentrations. A 25-minute gradient method was used with a 25-µL injection volume and a 1 mL/min flow rate at 30 ^o^C (EPA-NERL: 300.0).

*DNA extraction and sequencing*

Samples for DNA extraction were thawed for ~ 1hr, suspended in 20mL of chilled PBS /0.1% Na-pyrophosphate solution, then vortexed for 1 min. The suspended fraction was decanted to a fresh tube and remaining sand was dried in a convection oven at 70^o^C for 72hrs to determine the dry mass. Decanted liquid was centrifuged for 15 minutes at 7000xg and 10^o^C, supernatant was discarded and the remaining pellet was stored at -80^o^C. DNA was extracted from pellets using the MoBio PowerSoil kit in plate format (MoBio Laboratories, Inc., Carlsbad, CA). Pellets were re-suspended in 750µL of C1 bead solution and transferred to a 96-well bead beating plate*.* DNA was extracted following manufacturer’s instructions with the addition of a 2 hour proteinase-K incubation at 55^o^C prior to bead-beating to facilitate cell lysis. DNA was extracted from Sterivex filters in a 96-well plate format using the MoBio PowerSoil kit as described above. Extraction was done on a Eppendorf epMotion 5075 (Eppendorf AG, Hamburg, GE), with the bead beating step using a Retsch MM400 plate shaker (Retsch, Inc., Newton, PA).

PCR amplification of the V4 region of the 16S rRNA gene was performed using the protocol developed by the Earth Microbiome Project (http://press.igsb.anl.gov/earthmicrobiome/emp-standard-protocols/16s/), and described in Caporaso et al (2012), with the exception that the twelve base barcode sequence was included in the forward primer. Amplicons were sequenced on an Illumina MiSeq using the 300 cycle MiSeq Reagent Kit v2 (http://www.illumina.com/) according to manufacturer’s instructions.

*Sequence Analysis*

Sequence analysis was performed using QIIME software (Caporaso *et al.*, 2010). Pair-end reads were merged and chimeric sequences were identified and removed using USEARCH. Operational taxonomic units (OTUs) were chosen using open reference OTU picking with 97% identity threshold in conjunction with sequences obtained from samples at the same location with identical preparation and sequenced on the same sequencing run. Taxonomy was assigned using the SILVA reference database (Pruesse *et al.*, 2007). Chloroplast sequences were removed, yielding samples with a minimum of 1 sequence and a maximum of 40,819 sequences. Samples containing less than 987 sequences were removed, and all others were rarefied to 987 sequences.

*Null Modeling*

We implemented null modeling methodology developed by Stegen et al. (2013;2015) using R software ([http://cran.r-project.org/](http://cran.r-project.org/)) to disentangle microbial community assembly processes. The approach uses turnover among communities to infer the strength of selection, requiring a phylogenetic signal in data that indicates more closely related taxa are more ecologically similar than distantly related taxa (Losos, 2008). This effect has been previously demonstrated for Hanford Site 300A subsurface communities (Stegen *et al.*, 2013). To evaluate the strength of selection, pairwise phylogenetic turnover between communities was calculated using the mean-nearest-taxon-distance (βMNTD) metric (Webb *et al.*, 2008;Fine and Kembel, 2011) in the R package ‘picante.’ For comparison, a null distribution of βMNTD values was generated by calculating pairwise βMNTD values from 999 randomizations, in which species were shuffled across phylogenetic tips. Communities were evaluated for significantly less turnover than expected (βNTI < -2, homogeneous selection) or more turnover than expected (βNTI > 2, variable selection) by comparing observed βMNTD values to the mean of the null distribution – and normalizing by its standard deviation – to yield βNTI (Stegen *et al.*, 2012).

Pairwise community comparisons that did not deviate from the null βMNTD distribution were evaluated for the influences of dispersal limitation and homogenizing dispersal by calculating the Raup-Crick metric extended to account for species relative abundances (RC_bray_), as per Stegen et al. (2013;2015). The global species pool for null realizations of Bray-Curtis dissimilarities consisted of all OTUs occurring more than once in our dataset plus those occurring once in groundwater and river water samples. Observed Bray-Curtis dissimilarities were compared to the null distribution to derive RC_bray_ according to Stegen et al. (2013;2015). RC_bray_ values > 0.95 were assumed to indicate prevailing dispersal limitation with RC_bray_ values < -0.95 indicating homogenizing dispersal and RC_bray_ between -0.95 and 0.95 indicating no dominant assembly process. The noted inferences derived from both βNTI and RC_bray_ have previously been shown to be robust (Dini-Andreote *et al.*, 2015;Stegen *et al.*, 2015)

*Univariate Analysis*

Variables exhibiting non-normal distributions were log transformed prior to regression analysis. Variation in temperature and species richness through time were assessed with quadratic regression models. Species richness (defined as the number of species observed in a sample) was assessed for correlations with porewater temperature, chloride concentration, and NPOC content using linear or quadratic regression models, as appropriate. Differences in chloride and NPOC concentrations before and after a shift in hydrology at July 22 were assessed with one-sided Mann Whitney *U* tests.

In attached communities, Raz:ATP was assessed through time with linear regression, and correlations between Raz:ATP and temperature, chloride concentration, and NPOC content as well as with the relative abundance of *Betaproteobacteria* and *Thaumarchaeota* were determined with linear regressions. Finally, correlations of *Betaproteobacteria* and *Thaumarchaeota* with day of year as well as with porewater characteristics were assessed with linear or quadratic regressions. The relative abundance of *Betaproteobacteria* and *Thaumarchaeota* when Cl^-^ were below versus than above the maximum concentration observed in the river samples (5.16e-05 M/L) were assessed with one-sided Mann Whitney *U* tests.

*Multivariate Statistical Approaches*

Bray-Curtis distances between samples and species richness within each sample were derived using the QIIME pipeline (Caporaso *et al.*, 2010). Variation in community structure between attached and planktonic communities assessed through time by PERMANOVA in QIIME. SIMPER was conducted across attached and planktonic communities and within attached and planktonic communities separately. Moreover, SIMPER was conducted across time periods of high and low species richness within nearshore attached and planktonic communities to accommodate temporal trends evident in the data. Time periods were defined by visual examination of species richness plots through time in each group, as samples collected between May 20 and September 3 in planktonic samples and between June 30 and November 25 in attached samples. Finally, community composition in attached and planktonic communities was described with bi-directional non-metric multidimensional scaling analysis on Bray-Curtis distance matrices using the ‘vegan’ package in *R*. Porewater characteristics were fit to NMDS plots with and without stratifying by time to determine the influence of environmental variables on community structure at short and long timescales (999 permutations).

Pairwise differences in species richness and associated βNTI values were compared between and within attached and planktonic communities using Mantel tests in the ‘vegan’ package (999 permutations). Samples with βNTI values of >-2 and <2 were further analyzed by comparing their RC_bray_ values to species richness differences using Mantel tests. Lastly, the average relative abundance of taxonomic groups identified by SIMPER were compared to associated βNTI and RC_bray_ values.

**Supplemental Figures**

**Figure S1** Our sampling period encompassed pronounced seasonal variation in hydrology during 2014, with Columbia River stage ranging from 104.34 m to 107.23 m. Sampling dates are denoted with vertical dashed lines.

**Figure S2** Species richness in attached (X’s) and planktonic (triangles) communities was positively correlated with temperature. Lines denote linear and quadratic regression results for attached and planktonic communities, respectively.

**Supplemental Tables**

**Table S1** We measured physicochemical parameters in three geographic zones at the Hanford 300A site, sampling river, nearshore, and inland environments. Mean values of these parameters over the duration of our sampling period are reported for each zone in Table S1, with standard deviations listed in parentheses.

**Table S2** Dates and number of samples collected.

**Table S3** Composition of major taxa in attached and planktonic communities.

**Table S4** Physicochemical characteristics were fit to non-metric multidimensional scaling (NMDS) analysis of Bray-Curtis distances in attached and planktonic communities. Analyses were conducted both with and without stratifying permutations within the date of sample collection. *R^2^* values for significant parameters (P < 0.05) in each analysis are listed in Table S4. Data are also present in Figure 3 in the main text.

**Table S5** SIMPER analysis was conducted to identify species driving dissimilarity in attached and planktonic communities in periods of high vs. low richness. Species identifiers and associated taxonomy are present in Table S5 for all significant species (P < 0.05). For Table, please refer to supplemental Excel file.

**Table S6** The mean abundance (across two samples) of taxa identified by SIMPER analysis were compared to associated βNTI and RC_bray_ values (when -2 < βNTI < 2). Pearson’s *r* values greater than +/- 0.30 are listed in Table S6. A single asterisk denotes P < 0.05, double asterisks denote P < 0.01, and triple asterisks denote P < 0.001. ****

**Table S7** Factors correlating with the relative abundance of *Thaumarchaeota* and *Betaproteobacteria* was assessed using univariate linear regressions against physicochemical parameters. R^2^ values are presented in Table S7. Positive correlations are in bold italics, while negative correlations are in plain font. A single asterisk denotes P < 0.05, double asterisks denote P < 0.01, triple asterisks denote P < 0.001, and quadruple asterisks denote P < 0.0001.

**Table S8** Correlations of NPOC, Cl^-^, and temperature with community composition (Bray-Curtis distance matrices) were assessed using ADONIS. R^2^ values are presented in Table S8. A single asterisk denotes P < 0.05 and double asterisks denote P < 0.01.

**References**

Caporaso, J.G., Kuczynski, J., Stombaugh, J., Bittinger, K., Bushman, F.D., Costello, E.K., Fierer, N., Pena, A.G., Goodrich, J.K., and Gordon, J.I. (2010). QIIME allows analysis of high-throughput community sequencing data. *Nature methods* 7**,** 335-336.

Caporaso, J.G., Lauber, C.L., Walters, W.A., Berg-Lyons, D., Huntley, J., Fierer, N., Owens, S.M., Betley, J., Fraser, L., and Bauer, M. (2012). Ultra-high-throughput microbial community analysis on the Illumina HiSeq and MiSeq platforms. *The ISME journal* 6**,** 1621-1624.

Dini-Andreote, F., Stegen, J.C., Van Elsas, J.D., and Salles, J.F. (2015). Disentangling mechanisms that mediate the balance between stochastic and deterministic processes in microbial succession. *Proceedings of the National Academy of Sciences* 112**,** E1326-E1332.

Fine, P.V.A., and Kembel, S.W. (2011). Phylogenetic community structure and phylogenetic turnover across space and edaphic gradients in western Amazonian tree communities. *Ecography* 34**,** 552-565.

Hedges, J.I., and Stern, J.H. (1984). Carbon and nitrogen determinations of carbonate‐containing solids. *Limnology and Oceanography* 29**,** 657-663.

Losos, J.B. (2008). Phylogenetic niche conservatism, phylogenetic signal and the relationship between phylogenetic relatedness and ecological similarity among species. *Ecology letters* 11**,** 995-1003.

Pruesse, E., Quast, C., Knittel, K., Fuchs, B.M., Ludwig, W., Peplies, J., and Glöckner, F.O. (2007). SILVA: a comprehensive online resource for quality checked and aligned ribosomal RNA sequence data compatible with ARB. *Nucleic acids research* 35**,** 7188-7196.

Stegen, J.C., Lin, X., Fredrickson, J.K., Chen, X., Kennedy, D.W., Murray, C.J., Rockhold, M.L., and Konopka, A. (2013). Quantifying community assembly processes and identifying features that impose them. *The ISME journal* 7**,** 2069-2079.

Stegen, J.C., Lin, X., Fredrickson, J.K., and Konopka, A.E. (2015). Estimating and mapping ecological processes influencing microbial community assembly. *Frontiers in microbiology* 6.

Stegen, J.C., Lin, X., Konopka, A.E., and Fredrickson, J.K. (2012). Stochastic and deterministic assembly processes in subsurface microbial communities. *The ISME journal* 6**,** 1653-1664.

Webb, C.O., Ackerly, D.D., and Kembel, S.W. (2008). Phylocom: software for the analysis of phylogenetic community structure and trait evolution. *Bioinformatics* 24**,** 2098-2100.
